# FragNet, a Contrastive Learning-Based Transformer Model for Clustering, Interpreting, Visualizing, and Navigating Chemical Space

**DOI:** 10.3390/molecules26072065

**Published:** 2021-04-03

**Authors:** Aditya Divyakant Shrivastava, Douglas B. Kell

**Affiliations:** 1 Department of Computer Science and Engineering, Nirma University, Ahmedabad 382481, India; 17bit014@nirmauni.ac.in; 2 Department of Biochemistry and Systems Biology, Institute of Systems, Molecular and Integrative Biology, University of Liverpool, Crown St., Liverpool L69 7ZB, UK; 3 Novo Nordisk Foundation Centre for Biosustainability, Technical University of Denmark, Building 220, Kemitorvet, 2800 Kgs Lyngby, Denmark; 4 Mellizyme Ltd., Liverpool Science Park, IC1, 131 Mount Pleasant, Liverpool L3 5TF, UK

**Keywords:** deep learning, artificial intelligence, generative methods, chemical space, neural networks, transformers, attention, cheminformatics

## Abstract

The question of molecular similarity is core in cheminformatics and is usually assessed via a *pairwise* comparison based on vectors of properties or molecular fingerprints. We recently exploited variational autoencoders to embed 6M molecules in a chemical space, such that their (Euclidean) distance within the latent space so formed could be assessed within the framework of the entire molecular set. However, the standard objective function used did not seek to manipulate the latent space so as to cluster the molecules based on any perceived similarity. Using a set of some 160,000 molecules of biological relevance, we here bring together three modern elements of deep learning to create a novel and disentangled latent space, viz transformers, contrastive learning, and an embedded autoencoder. The effective dimensionality of the latent space was varied such that clear separation of individual types of molecules could be observed within individual dimensions of the latent space. The capacity of the network was such that many dimensions were not populated at all. As before, we assessed the utility of the representation by comparing clozapine with its near neighbors, and we also did the same for various antibiotics related to flucloxacillin. Transformers, especially when as here coupled with contrastive learning, effectively provide one-shot learning and lead to a successful and disentangled representation of molecular latent spaces that at once uses the entire training set in their construction while allowing “similar” molecules to cluster together in an effective and interpretable way.

## 1. Introduction

The relatively recent development and success of “deep learning” methods involving “large”, artificial neural networks (e.g., [1,2,3,4]) has brought into focus a number of important features that can serve to improve them further, in particular with regard to the “latent spaces” that they encode internally. One particular recognition is that the much greater availability of unlabeled than labelled (supervised learning) data can be exploited in the creation of such deep nets (whatever their architecture), for instance in variational autoencoders [5,6,7,8,9], or in transformers [10,11,12].

A second trend involves the recognition that the internal workings of deep nets can be rather opaque, and especially in medicine there is a desire for systems that explain precisely the features they are using in order to solve classification or regression problems. This is often referred to as “explainable AI” [13,14,15,16,17,18,19,20,21,22]. The most obviously explainable networks are those in which individual dimensions of the latent space more or less directly reflect or represent identifiable features of the inputs; in the case of images of faces, for example, this would occur when the value of a feature in one dimension varies smoothly with and thus can be seen to represent, an input feature such as hair color, the presence or type of spectacles, the presence or type of a moustache, and so on [23,24,25,26]. This is known as a disentangled representation (e.g., [27,28,29,30,31,32,33,34,35,36,37,38]). To this end, it is worth commenting that the ability to generate more or less realistic facial image structures using orthogonal features extracted from a database or collection of relevant objects that can be parametrized has been known for some decades [39,40,41,42].

Given an initialization, the objective function of a deep network necessarily determines the structure of its latent space. Typical variational autoencoders seek to minimize the evidence lower bound (ELBO) of the Kulback–Leibler (KL) divergence between the desired and calculated output distributions [43,44,45,46], although many other variants with different objective functions have been suggested (e.g., [45,47,48,49,50,51,52,53,54]). However, a third development is the recognition that training with such unlabeled data can also be used to optimize the (self-) organization of the latent space itself. A particular objective of one kind of self-organization is one in which individual inputs are used to create a structure in which similar input examples are also closer to each other in the latent space; this is commonly referred to as self-supervised [12,55,56,57], or contrastive [58,59,60,61,62,63,64,65,66], learning. In image processing this is often performed by augmenting training data with rotated or otherwise distorted versions of a given image, which then retain the same class membership or “similarity” despite appearing very different [61,67,68,69]. Our interests here are in molecular similarity.

### Molecular Similarity

Molecular (as with any other kind of) similarity [70,71,72] is a somewhat elusive but, importantly, unsupervised concept in which we seek a metric to describe, in some sense, how closely related two entities are to each other from their structure or appearance alone. The set of all small molecules of possible interest for some purpose, subject to constraints such as commercial availability [73], synthetic accessibility [74,75], or “drug-likeness” [76,77], is commonly referred to “chemical space”, and it is very large [78,79,80,81,82,83,84,85,86,87,88,89,90,91,92,93,94,95,96,97]. In cheminformatics the concept of similarity is widely used to prioritize the choice of molecules “similar” to an initial molecule (usually a “hit” with a given property or activity in an assay of interest) from this chemical space or by comparison with those in a database, on the grounds that “similar” molecular structures tend to have “similar” bioactivities [98].

The problem with this is that the usual range of typical metrics of similarity, whether using molecular fingerprints or vectors of the values of property descriptors, tend to give quite different values for the similarity of a given pair of molecules (e.g., [99]). In addition, and importantly, such pairwise evaluations are done individually, and their construction takes no account of the overall structure and population of the chemical space.

#### Deep Learning for Molecular Similarity

In a recent paper [5], we constructed a subset of chemical space using six million molecules taken from the ZINC database [100] (www.zincdocking.org/, accessed on 28 February 2021), employing a variational autoencoder to construct the latent space used to represent 2D chemical structures. The latent space is a space between the encoder and the decoder with a certain dimensionality D such that the position of an individual molecule in the latent space, and hence the chemical space is simply represented by a D-dimensional vector. A brief survey [5] implied that molecules near each other in this chemical space did indeed tend to exhibit evident and useful structural similarities, though no attempt was made there either to exploit contrastive learning or to assess degrees of similarity systematically. Thus, it is correspondingly unlikely that we had optimized the latent space from the points of view of either optimal feature extraction or explainability.

The most obvious disentanglement for small molecules, which is equivalent to feature extraction in images, is surely the extraction of molecular fragments or substructures, that can then simply be “bolted together” in different ways to create any other larger molecule(s). Thus, it is reasonable that a successful disentangled representation would involve the principled extraction of useful substructures (or small molecules) taken from the molecules used in the training. In this case we have an additional advantage over those interested in image processing, because we have other effective means for assessing molecular similarity, and these do tend to work for molecules with a Tanimoto similarity (TS) greater than about 0.8 [99]; such molecules can then be said to be similar, providing positive examples for contrastive learning (although in this case we use a different encoding strategy). Pairwise comparisons returning TS values lower than say 0.3 may similarly be considered to represent negative examples.

Nowadays, transformer architectures (e.g., [3,11,12,101,102,103,104,105,106,107,108,109,110]) are seen as the state of the art for deep learning of the type of present interest. As per the definition of the contrastive learning framework mentioned in [61,66], we add an extra autoencoder in which the encoder behaves as a projection head. The outputs of the transformer encoder, which we regard as representations, are to be of a higher dimension. Consequently, it can still take a relatively large computational effort to compute the similarity between the representations. To this end, we add a simple encoder network that maps the representations to a lower dimensional latent space on which the contrastive loss is computationally easier to define. Then, to convert the latent vector again to the appropriate representations to feed into the transformer decoder network, we add a simple decoder network.

In sum, therefore, it seemed sensible to bring together both contrastive learning and transformer architectures so as to seek a latent space optimized for substructure or molecular fragment extraction. Consequently we refer to this method as FragNet. The purpose of the present paper is to describe our implementation of this, recognizing that SMILES strings represent sequences of characters just as do the words used in natural language processing. During the preparation of this paper, a related approach also appeared [111] but used graphs rather than a SMILES encoding of the structures.

## 2. Results

**Figure 1:**
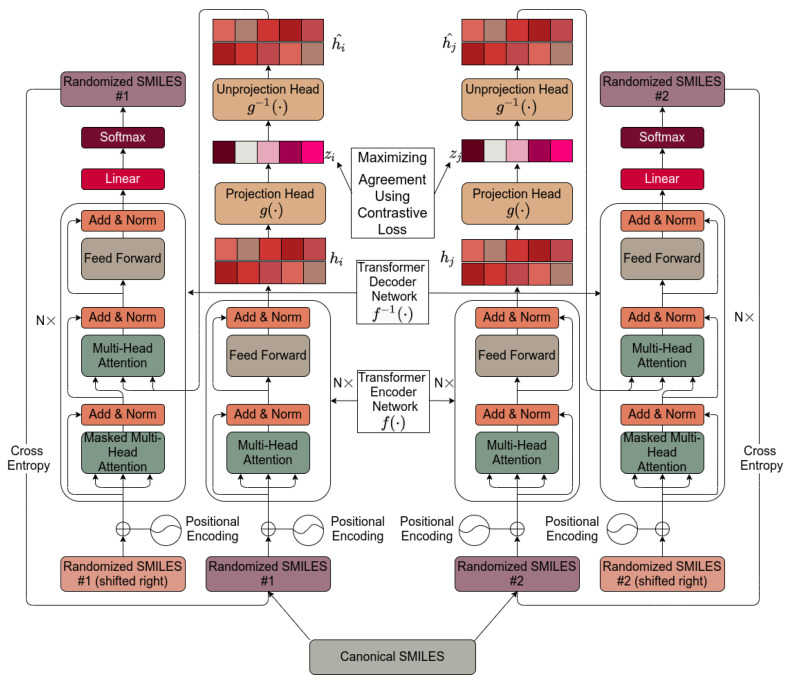
The transformer-based architecture used in the present work. The internals are described in Section 4.

**Scheme 1:**
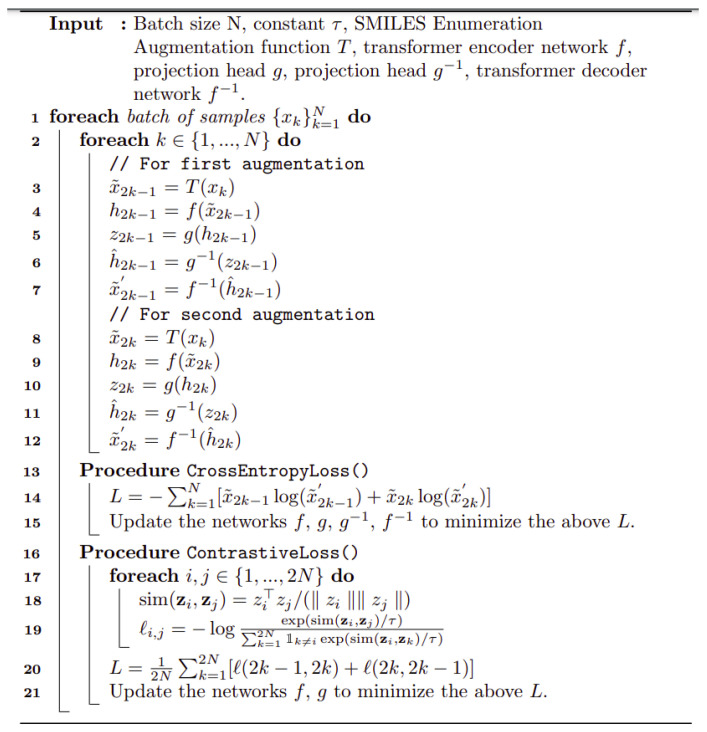
Pseudocode for the transformer algorithm as implemented here.

Figure 1 shows the basic architecture chosen, essentially as set down by [112]. It is based on [112] and is described in detail in Section 4. Pseudocode for the algorithm used is given in Scheme 1.

**Figure 2:**
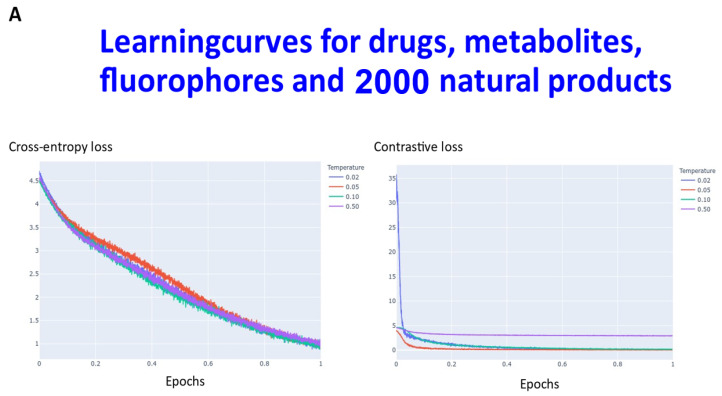

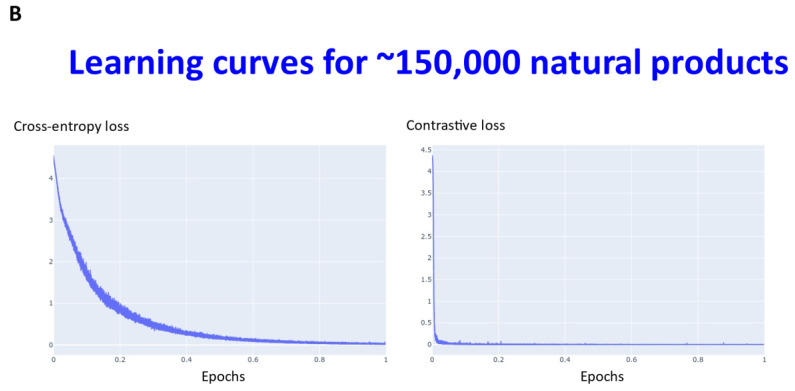
Learning curve for training our transformers on (**A**) drugs, metabolites, fluorophores, and 2000 natural products, and (**B**) a full set of natural products. Because the transformer is effectively a one-shot learner, and the batch size varied, the abscissa is shown as a single epoch. The batch size was varied, as described in Section 4, and was (**A**) 50 (latent space of 64 dimensions) and (**B**) 20 (latent space of 256 dimensions), leading to an actual number of batches of (**A**) 92 and (**B**) 7500.

Transformers are computationally demanding (our largest network had some 4.68 M parameters), and so (as described in Section 4) instead of using 6M ZINC molecules (that the memory available in our computational resources could not accommodate), we studied datasets consisting overall of ~160,000 natural products, fluorophores, endogenous metabolites, and marketed drugs (the dataset is provided in [113]). We compared contrastive learning with the conventional objective function in which we used the evidence lower bound of the KL divergence. The first dataset (Materials and Methods) consisted of ~5000 (actually 4643) drugs, metabolites, and fluorophores, and 2000 UNPD natural products molecules, while the second consisted of the full set of ~150 k natural products. “Few-shot” learning (e.g., [114,115,116]) means that only a very small number of data points are required to train a learning model, while “one-shot” learning (e.g., [117,118,119,120,121]) involves the learning of generalizable information about object categories from a single related training example. In appropriate circumstances, transformers can act as few-shot [3,122,123], or (as here) even one-shot learners [124,125]. We thus first compared the learning curves of transformers trained using cross entropy versus those trained using contrastive loss (Figure 2). In each case, the transformer-based learning essentially amounts to one-shot learning, especially for the contrastive case, and so the learning curve is given in terms of the effective fraction of the training set. We note that recent studies happily imply that large networks of the present type are indeed surprisingly resistant to overtraining [126]. In Figure 2A the optimal temperature used seemed to be 0.05 and this was used for the larger dataset (Figure 2B). The clock time for training an epoch on a single NVIDIA-V100-GPU system was ca. 30 s and 23 min for the two datasets illustrated in Figure 2A and Figure 2B, respectively.

**Figure 3:**
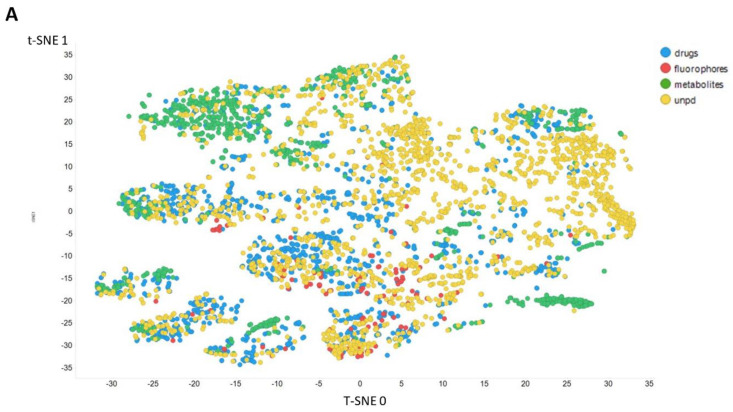

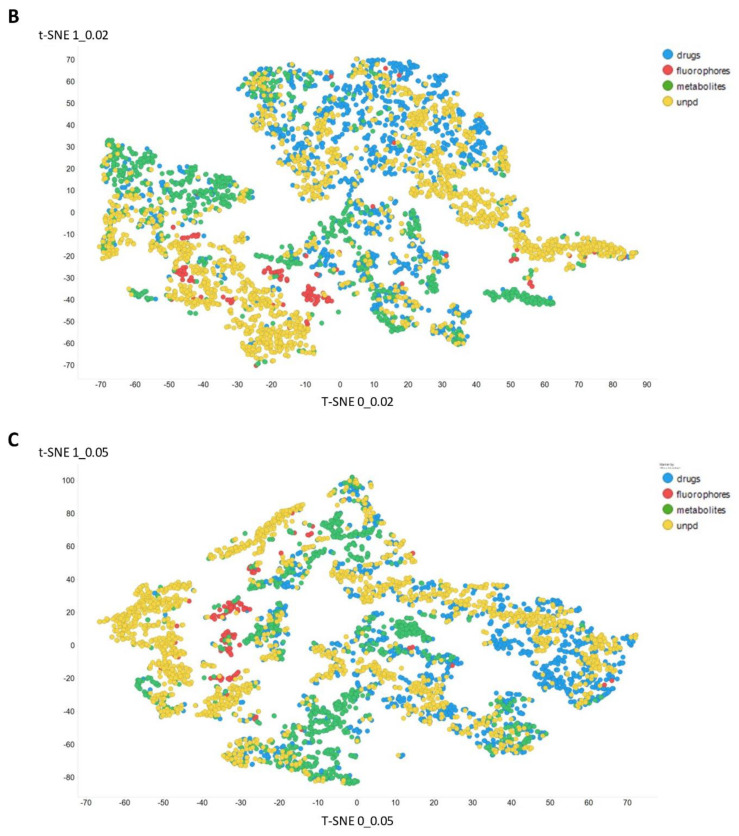

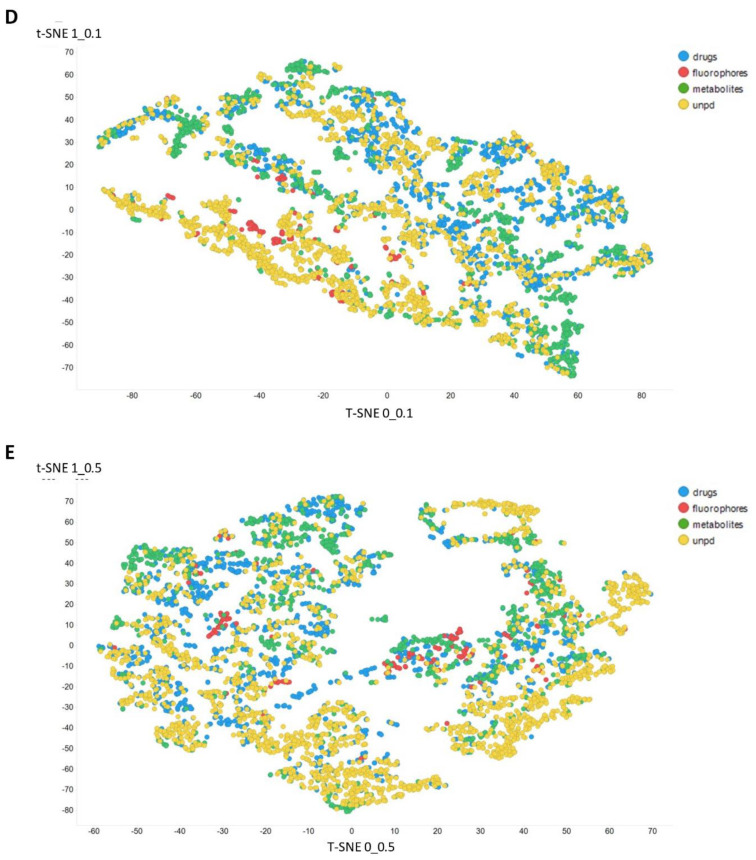
Effect of adjusting the temperature parameter in the contrastive learning loss on the distribution of molecules in the latent space as visualized via the t-SNE algorithm. For clarity, only a random subset of 2000 natural products is shown. (**A**) Learning based purely on the cross-entropy objective function. (**B**–**E**) The temperature scalar (as in [112]) was varied between 0.02 and 0.5 as indicated. (Reducing t below led to numerical instabilities.) All drugs, fluorophores, and Recon2 metabolites are plotted, along with a randomly chosen 2000 natural products (as in [113]).

Figure 3 gives an overall picture using t-SNE [127,128] of the dataset used. Figure 3A recapitulates that published previously, using standard VAE-type ELBO/K-L divergence learning alone, while panels Figure 3B–E show the considerable effect of varying the temperature scalar (as in [112]).

It can clearly be seen from Figure 3B–E that as the temperature was increased in the series 0.02, 0.05, 0.1, and 0.5, the tightness and therefore the separability of the clusters progressively decreased. For instance, by mainly looking at the fluorophores (red colors) in the plotted latent space for each of the four temperatures, the separability as well as tightness of the cluster was best for the 0.02 and 0.05 temperatures. Later, as the temperature increased to 0.1, the data points became more dispersed, and finally at a temperature of 0.5, the data points were the most dispersed. Therefore, we suggest that (while the effect is not excessive) the reduced temperature may lead to the data points being more tightly clustered. However, the apparent dependency is not linear.

**Figure 4:**
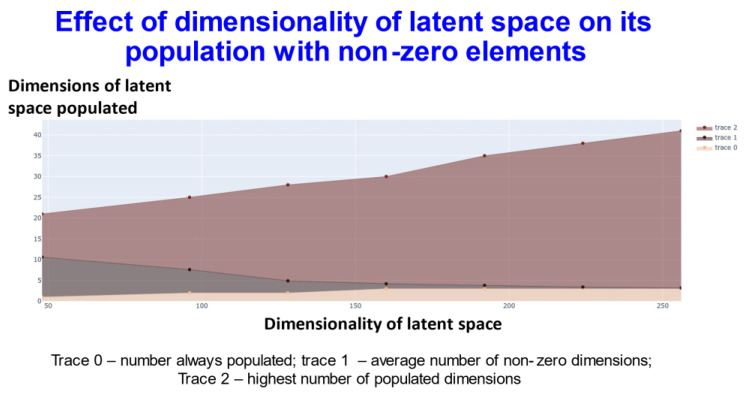
Relationship between the extent of population of different dimensions and the dimensionality of the latent space using transformers with contrastive learning.

We also varied the number of dimensions used in the latent space, which served to provide some interesting insights into the effectiveness of the disentanglement and the capacity of the transformer (Figure 4).

In Figure 4, trace 0 means that the elements of this number of dimensions was always nonzero. In other words, for every molecule, the value of at least that number of dimensions (the value on the *y*-axis) will be always non zero. Thus, for the 256-dimensional latent space three dimensions were always non-zero). Trace1 means the average of the number of dimensions that were non zero for the dataset. Finally trace2 gives the highest number of dimensions recorded as populated for that specific dimensional latent space. This shows (and see below) that while GPU memory requirements meant that we were limited to a comparatively small number of molecules in our ability to train a batch of molecules, the capacity of the network was very far from being exceeded, and in many cases some of the dimensions were not populated with non-zero values at all. At one level this might be seen as obvious: if we have 256 dimensions *and each could take only two values*, there are 2^256^ positions in this space (~10^77^). This large dimensionality at once explains the power and the storage capacity of large neural networks of this type.

**Figure 5:**
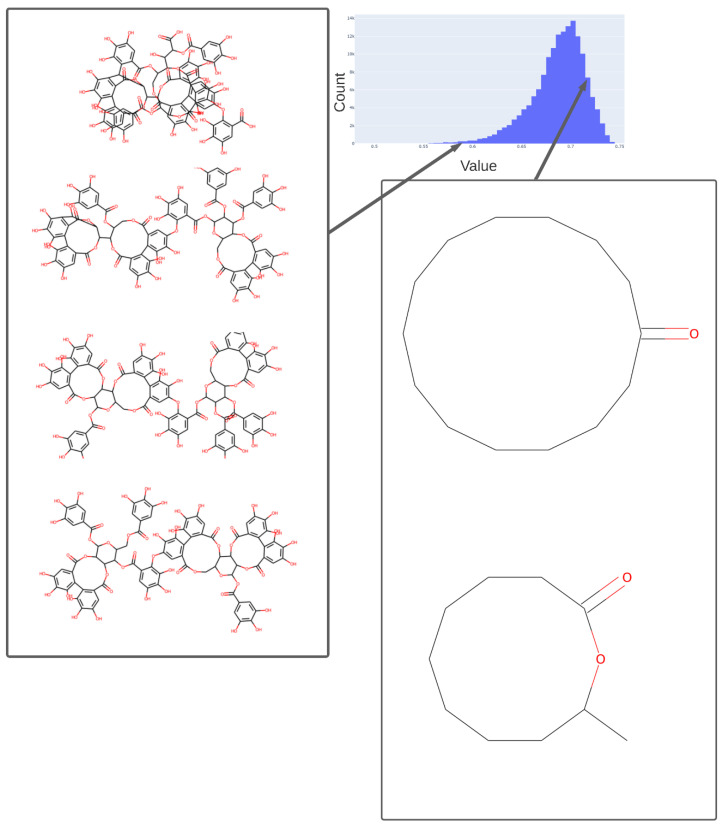
Values adopted in dimension 254 of the trained 256-D transformer, showing the values of various tri-hydroxy-benzene-containing compounds (left) ca. 0.59 and two lactones (ca. 0.73). The arrows indicate the bins (0.58, 0.73) in the histogram of values in this dimension from which the representative molecules shown were taken.

**Figure 6:**
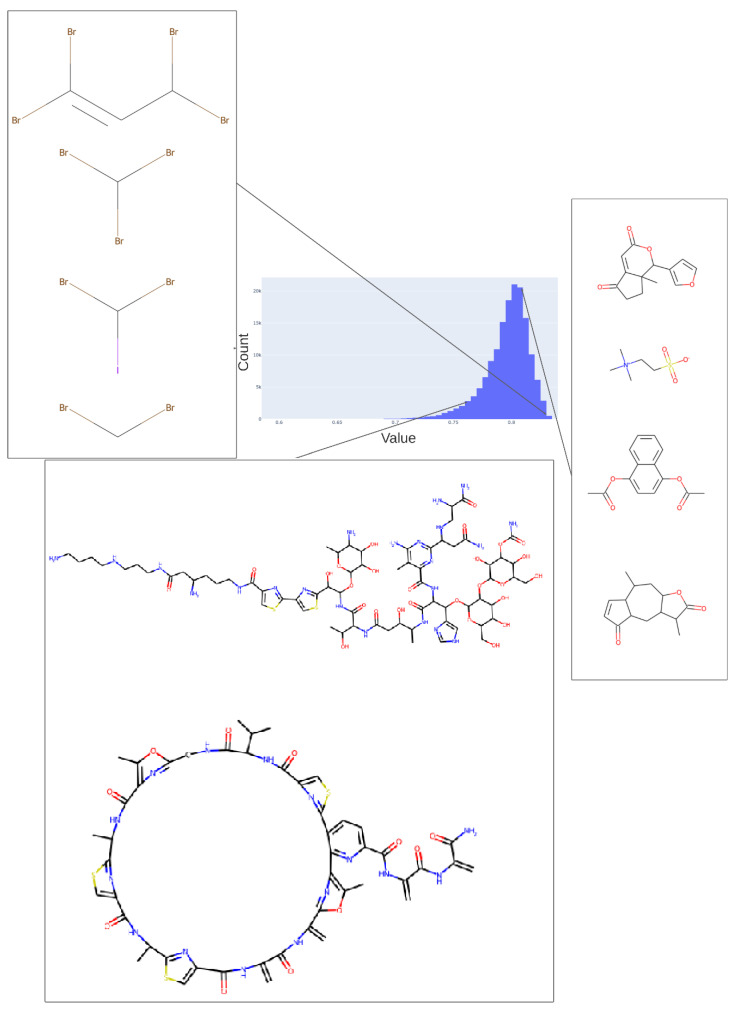
Values adopted in dimension 182 of the trained 256-D transformer, showing the values of various halide-containing (~0.835) and other molecules. As in Figure 5, we indicate the bins in the histogram of values (0.76, 0.81, 0.83) in this dimension from which the representative molecules shown were taken.

**Figure 7:**
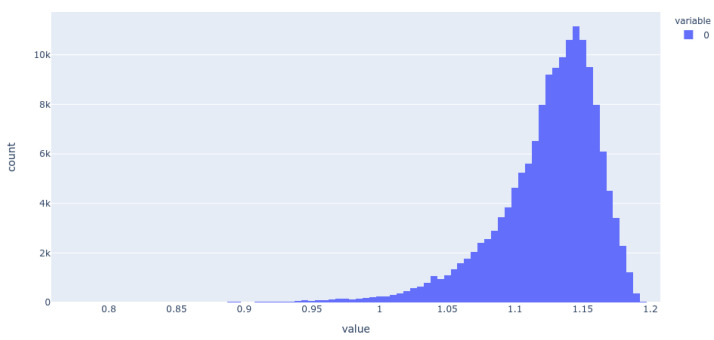
Histogram of the population of dimension 25 for the 256-D dataset. It is evident that most molecules adopt only a small range of non-zero values in this dimension.

We illustrate this further by showing the population of just three of the dimensions (for the 256-dimension case), viz dimensions 254 (Figure 5), 182 (Figure 6), and 25 (Figure 7) (these three were always populated with non-zero values).

**Figure 8:**
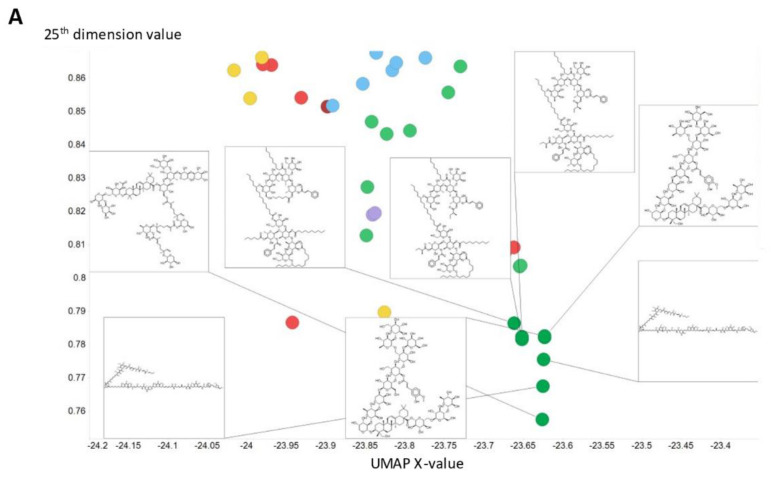

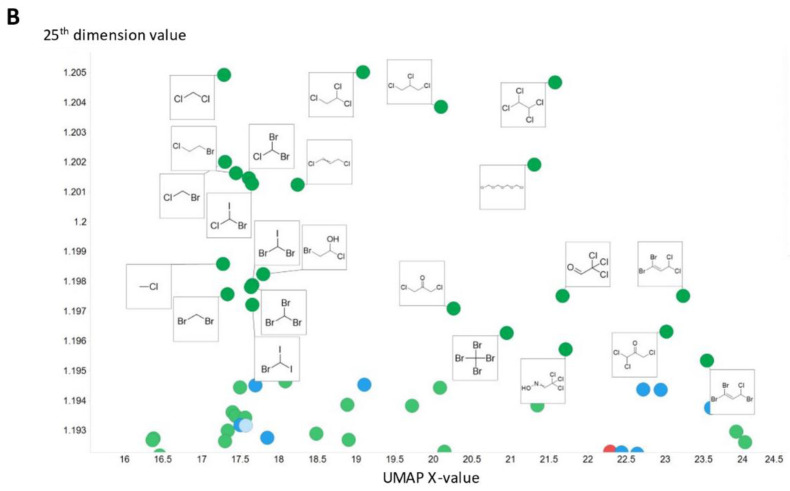
Effective disentanglement of molecular features into individual dimensions, using the indicated values of 25th dimension of the latent space of the 2nd dataset. In this case we used a latent space of 256 dimensions and a temperature t of 0.05. (**A**) Trihydroxycyclohexane derivatives, (**B**) halide-containing moieties.

To illustrate in more detail the effectiveness of the disentanglement, we illustrated a small fraction of the values of the 25th dimension alone, as plotted against a UMAP [129,130] X-coordinate. Despite the *tiny* part of the space involved (shown on the *y*-axis), it is clear that this dimension alone has extracted features that involve tri-hydroxylated cyclohexane- (Figure 8A) or halide-containing moieties (Figure 8B).

Another feature of this kind of chemical similarity analysis involves picking a molecule of interest and assessing what is “near” to it in the high-dimensional latent space, as judged by conventional measures of vector distance. We variously used the cosine or the Euclidean distance. As before [5], we chose clozapine as our first “target” molecule and used it to illustrate different feature of our method.

**Figure 9:**
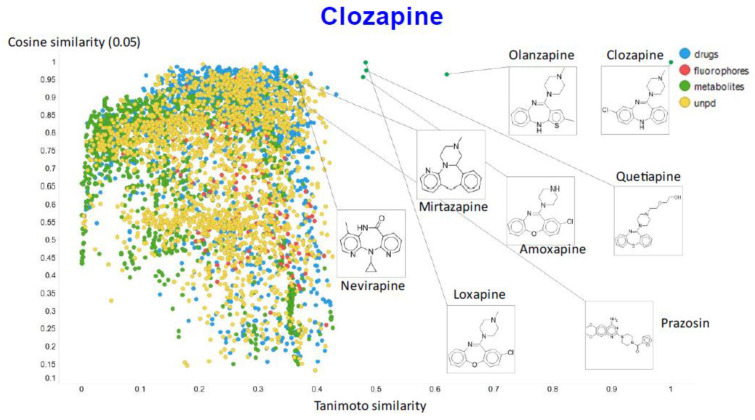
Relationship between cosine similarity and Tanimoto similarity for clozapine in our chemical space, using a temperature of 0.05.

Figure 9 illustrates the relationship (using a temperature factor of 0.05) between the cosine similarity and the Tanimoto similarity for clozapine (using RDKit’s RDKfingerprint encoding (https://www.rdkit.org/docs/source/rdkit.Chem.rdmolops.html, accessed on 28 February 2021).

It is clear that (i) very few molecules showed up as being similar to clozapine in Tanimoto space, while prazosin (which competes with it for transport [131]) had a high cosine similarity despite having a very low Tanimoto similarity. In particular, none of the molecules with a high Tanimoto similarity had a low cosine similarity, indicating that our method does recognize molecular similarities effectively.

**Figure 10:**
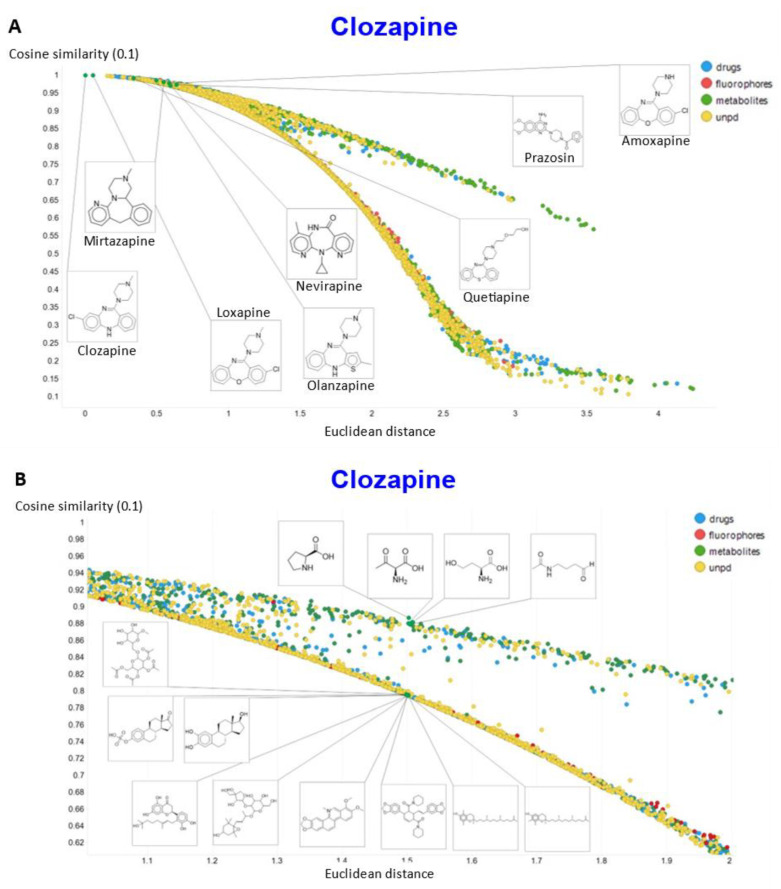
Relationship between cosine similarity and Euclidean distance for clozapine in our chemical space using a temperature of 0.1. (**A**) Overview. (**B**) Illustration of molecules in the bifurcation.

To show other features, Figure 10A shows the plots of the cosine similarity against the Euclidean distance; they were tolerably well correlated, with an interesting bifurcation, implying that the cosine similarity is probably to be preferred. This is because a zoomed-in version (Figure 10B) shows that the two sets of molecules with a similar Euclidean distance around 1.5 really are significantly different from each other between the two sets, where the cosine similarities also differ. By contrast, the molecules with a similar cosine similarity within a given arm of the bifurcation really are similar. The zooming in also makes it clear that the upper fork tends to have a significantly greater fraction of “Recon2” metabolites than does the lower fork, showing further how useful the disentangling that we have effected can be.

**Figure 11:**
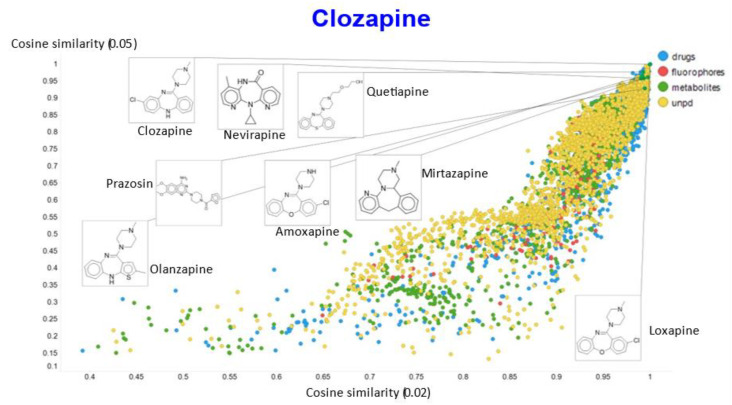
Relationship between cosine similarity for values of the temperature parameter of 0.05 and 0.02 for clozapine in our chemical space.

In a similar vein, varying the temperature scalar caused significant differences in the values of the cosine similarities for clozapine vs. the rest of the dataset (Figure 11).

**Figure 12:**
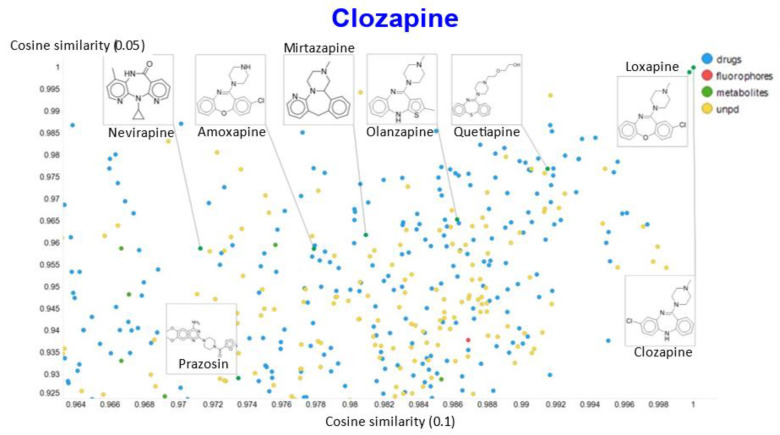
Relationship between cosine similarity for values of the temperature parameter of 0.05 and 0.1 for clozapine in our chemical space.

**Figure 13:**
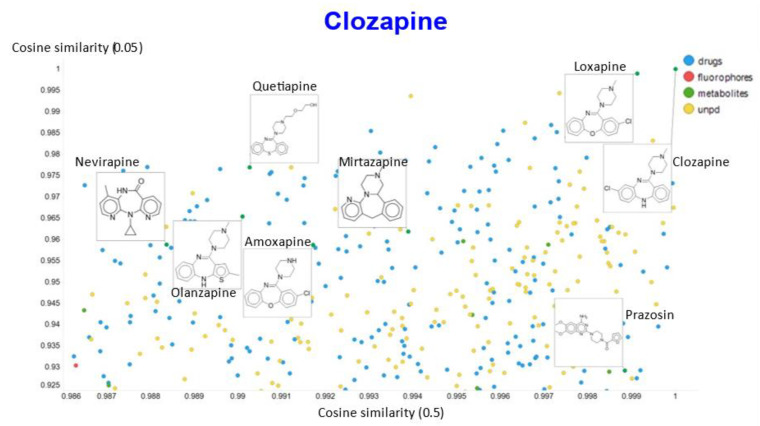
Relationship between cosine similarity for values of the temperature parameter of 0.05 and 0.5 for clozapine in our chemical space.

A similar plot is shown, at a higher resolution, for the cosine similarities with temperature scalars of 0.05 and 0.1 (Figure 12) and 0.05 vs. 0.5 (Figure 13). The closeness of clozapine to the other “apines”, as judged by cosine similarity, did vary somewhat with the value of the temperature. However, the latter value brings prazosin to be very close to clozapine, indicating the substantial effects that the choice of the temperature scalar can exert.

**Figure 14:**
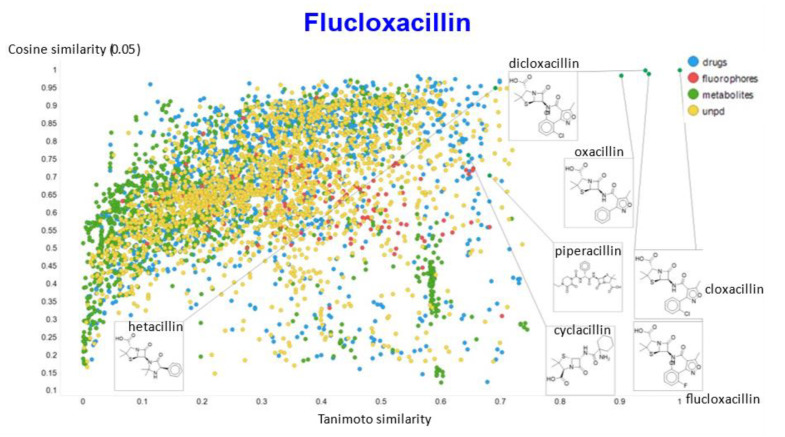
Relationship between cosine similarity and Tanimoto similarity (temperature = 0.05) for flucloxacillin in our chemical space.

**Figure 15:**
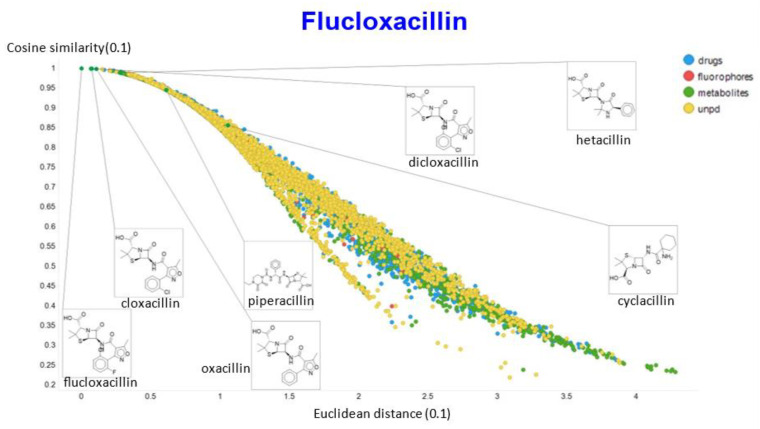
Relationship between cosine similarity and Euclidean distance for flucloxacillin in our chemical space, with a temperature parameter of 0.1.

**Figure 16:**
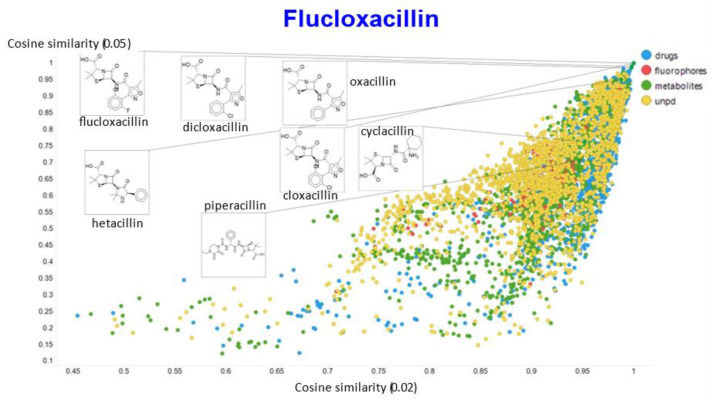
Relationship between cosine similarity for values of the temperature parameter of 0.05 and 0.02 for flucloxacillin in our chemical space.

**Figure 17:**
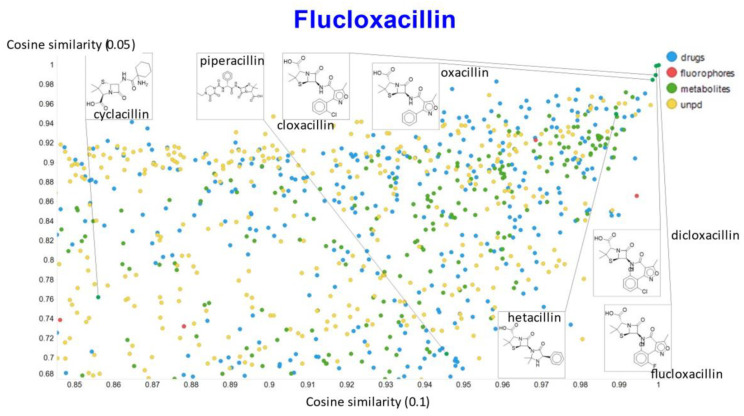
Relationship between cosine similarity for values of the temperature parameter of 0.05 and 0.1 for flucloxacillin in our chemical space.

**Figure 18:**
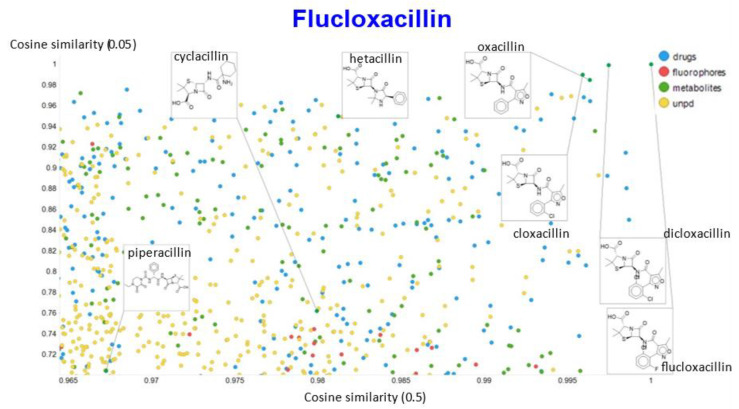
Relationship between cosine similarity for values of the temperature parameter of 0.05 and 0.5 for flucloxacillin in our chemical space.

A similar exercise was undertaken for “acillin”-type antibiotics based on flucloxacillin, with the results illustrated in Figure 14, Figure 15, Figure 16, Figure 17 and Figure 18.

In the case of flucloxacillin, the closeness of the other “acillins” varied more or less monotonically with the value of the temperature parameter. Thus for particular drugs of interest, it is likely best to fine tune the temperature parameter accordingly. In addition, the bifurcation seen in the case of clozapine was far less substantial in the case of flucloxacillin.

**Figure 19:**
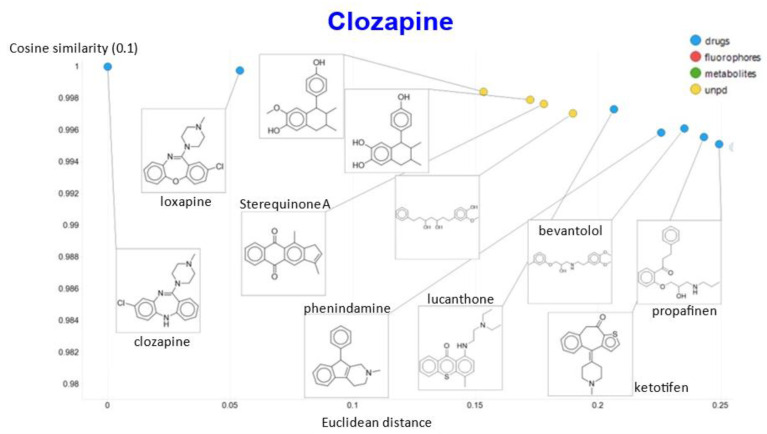
Molecules closest to clozapine when a temperature of 0.1 is used, as judged by both cosine similarity and Euclidean distance.

That the kinds of molecule that were most similar to clozapine do indeed share structural features is illustrated (Figure 19) for a temperature of 0.1 in both cosine and Euclidean similarities, where the 10 most similar molecules include six known antipsychotics, plus four related natural products that might be of interest to those involved in drug discovery.

**Figure 20:**
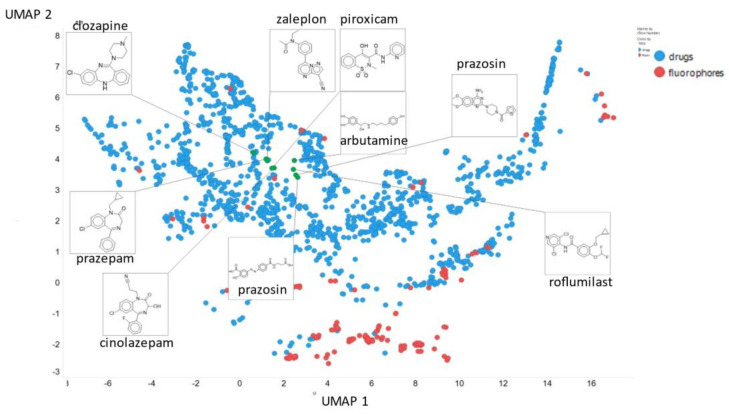
Positions of chlorpromazine, prazosin and some other molecules in UMAP space when the NT-Xent temperature factor is 0.1.

Finally, here we show (using for clarity drugs and fluorophores only (Figure 20)) the closeness of chlorpromazine and prazosin in UMAP space when the NT-Xent temperature factor is 0.1.

## 3. Discussion

The concept of molecular similarity is at the core of much of cheminformatics, on the simple grounds that structures that are more similar to each other tend to have more similar bioeffects, an elementary idea typically referred to as the “molecular similarity principle” (e.g., [98,132,133,134]). Its particular importance commonly comes in circumstances where one has a “hit” in a bioassay and wishes to select from a library of available molecules of known structure which ones to prioritize for further assays that might detect a more potent hit. The usual means of assessing molecular similarity are based on encoding the molecules as vectors of numbers based either on a list of measured or calculated biophysical or structural properties, or via the use of so-called molecular fingerprinting methods (e.g., [135,136,137,138,139,140,141,142]). We ourselves have used a variety of these methods in comparing the “similarity” between marketed drugs, endogenous metabolites and vitamins, natural products, and certain fluorophores [91,99,113,143,144,145,146,147,148].

At one level, the biggest problem with these kinds of methods is that all comparisons are done pairwise, and no attempt is thereby made to understand chemical space “as a whole”. In a previous paper [5], based in part on other “deep learning” strategies (e.g., [80,96,149,150,151,152,153,154,155,156,157,158,159]) we used a variational autoencoder (VAE) [6], to project some 6M molecules into a latent chemical space of some 192 dimensions. It was then possible to assess molecular similarity as a simple Euclidean distance.

A popular and more powerful alternative to the VAE is the transformer. Originally proposed by Vaswani and colleagues [11], transformers have come to dominate the list of preferred methods, especially those used with strings such as those involved in natural language processing [106,160,161,162,163]. Since chemical structures can be encoded as strings such as SMILES [164], it is clear that transformers might be used with success to attach problems involving small molecules, and they have indeed been so exploited (e.g., [10,12,104,165,166,167,168]). In the present work, we have adopted and refined the transformer architecture.

A second point is that in the previous work [5], we made no real attempt to manipulate the latent space so as to “disentangle” the input representations, and if one is to begin to understand the working of such “deep” neural networks it is necessary to do so. Of the various strategies available, those using contrastive learning [11,62,66,169,170,171] seem to be the most apposite. In contrastive learning, one informs the learning algorithm whether two (or more) individual examples come from the same of different classes. Since in the present case we do know the structures, it is relatively straightforward to assign “similarities”, and we used a SMILES augmentation method for this.

The standard transformer does not have an obvious latent space of the type generated by autoencoders (variational or otherwise). However, the SimCLR architecture admits its production using one of the transformer heads. To this end, we added a simple autoencoder to our transformer such that we could create a latent space with which to assess molecular similarity more easily. In the present case, we used cosine similarity, Tanimoto similarity, and Euclidean distance.

There is no “correct” answer for similarity methods, and as Everitt [172] points out, results are best assessed in relation to their utility. In this sense, it is clear that our method returns very sensible groupings of molecules that may be seen as similar by the trained chemical eye, and which in the cases illustrated (clozapine and flucloxacillin) clearly group molecules containing the base scaffold that contributes to both their activity and to their family membership (“apines” and “acillins”, respectively).

There has long been a general recognition (possibly as part of the search for “artificial general intelligence” (e.g., [173,174,175,176,177,178,179]) that one reason that human brains are more powerful than are artificial neural networks may be—at least in part—simply because the former contain vastly more neurons. What is now definitely increasingly clear is that very large transformer networks can both act as few-shot learners (e.g., [3,108]) and are indeed able to demonstrate extremely powerful generative properties, albeit within somewhat restricted domains. Even though the limitations on the GPU memory that we could access meant that we studied only some 160,000 molecules, our analysis of the contents of the largest transformer trained with contrastive learning indicated that it was nonetheless very sparsely populated. This both illustrates the capacity of these large networks and leads necessarily to an extremely efficient means of training.

Looking to the future, as more computational resources become available (with transformers using larger networks for their function), we can anticipate the ability to address and segment much larger chemical spaces, and to use our disentangled transformer-based representation for the encoding of molecular structures for a variety of both supervised and unsupervised problem domains.

## 4. Materials and Methods

We developed a novel hybrid framework by combining three things, namely transformers, an auto-encoder, and a contrastive learning framework. The complete framework is shown in Figure 1. The architecture chosen was based on the SimCLR framework of Hinton and colleagues [61,112], to which we added an autoencoder so as to provide a convenient latent space for analysis and extraction. Programs were written in PyTorch within an Anaconda environment. They were mostly run one GPU of a 4-GPU (NVIDIA V100) system. The dataset used included ~150,000 natural products [91,99,148], plus fluorophores [113], Recon2 endogenous human metabolites [143,144,146,147], and FDA-approved drugs [99,143,144,145], as previously described. Visualization tools such as t-SNE [127,128] and UMAP [129,130] were implemented as previously described [113]. The dataset was split into training and validation and test sets as described below.

We here develop a novel hybrid framework upon the contrastive learning framework using transformers. We explain the complete framework with each of the components as below:

### 4.1. Molecular SMILES Augmentation

Contrastive learning is all about uniting positive pairs and discriminating between negative pairs. The first objective is thus to develop an efficient way of determining positive and negative data pairs for the model. We adopted the SMILES enumeration data augmentation technique from Bjerrum [180], that any given canonical SMILES data example can generate multiple SMILES strings that basically represent the same molecule. We used this technique to sample two different SMILES strings x_i_ and x_j_ from every canonical SMILES string from the dataset, which we regarded as positive pairs.

### 4.2. Base Encoder

Once we received the augmented, randomized SMILES, they were added with their respective positional encoding. The positional encoding is a sine or cosine function defined according to the position of a token in the input sequence length. It is done in order also to take into consideration the order of the sequence. The next component of the framework is the encoder network that takes in the summation of the input sequence and its positional encoding and extracts the representation vectors for those samples. As stated by Chen and colleagues [112], there is complete freedom when it comes to the choice of architecture for the encoder network. Therefore, we used a transformer encoder network, which has in recent years become the state-of-the-art for language modelling tasks and has been subsequently significantly extended to chemical domains as well.

As set down in the original transformers paper, the transformer encoder basically comprises two sub-blocks. The first sub-block has a multi-head attention layer followed by a layer normalization layer. The first multi-head attention layer makes the model pay attention to the values at neighbors’ positions when encoding a representation for one particular position. Then, the layer normalization layer normalizes the sum of inputs obtained from the residual connection and the outputs of the multi-head attention layer. The second block consists of a feed forward network, one for every position. Then, similar to the previous case, layer norm is defined on the position-wise sum of the outputs from the feed forward layer and the residually connected output from the previous block.

The output of the transformer encoder network is an array of feature-embedding vectors which we call the representation (h_i_). The representation obtained from the network is of the dimension sequential length × d_model_. This means that the transformer encoder network generates feature embedding vectors for every position in the input sequence length. Normally, these transformer encoder network blocks are repeated N times and the output representation of one encoder is an input of another. Here, we employed 4 transformer encoder blocks.

### 4.3. Projection Head

The projection head is a simple encoder neural network to project the feature embedding representation vector of shape (input sequence length × d_model_) down to a lower dimension representation of shape (1 × d_model_). Here, we used an artificial neural network of 4 layers with the ReLu activation function. This gave an output projection vector z_i_, which was then used for defining the contrastive loss.

### 4.4. Contrastive Loss

As the choice of contrastive loss for our experiments, we used the normalized temperature-scaled cross entropy (NT-Xent) loss [64,112,181,182].

(1) $$L_{i,j}=\log\frac{\exp\left(\mathrm{sim}\left(z_{i},z_{j}\right)/\tau \right)}{\sum_{k=1}^{2N}𝟙\left\{k\neq i\right\}\exp\left(\mathrm{sim}\left(z_{i},z_{k}\right)/\tau \right)}$$

where z_i_ and z_j_ are positive pair projection vectors when two transformer models are run in parallel. 𝟙{k≠i} is a Boolean evaluating to 1 if k is not the same as i, and τ is the temperature parameter. Lastly, sim() is the similarity metric for estimating the similarity between z_i_ and z_j_. The idea behind using this loss function is that when sampling a sample batch of data of size N for training, each sample is augmented as per subsection “Section 4.1” and the total would then be 2N samples. Therefore, for every sample there is one other sample from the same canonical SMILES and 2N-2 other samples. Therefore, we considered for every sample one other sample generated from the same canonical SMILES as a positive pair and each of the other 2N-2 samples as a negative pair.

### 4.5. Unprojection Head

Unlike SimCLR or any other previous contrastive learning framework, we also opted to include a simple decoder network and then a transformer decoder network through which we also taught the model to generate a molecular SMILES representation whenever queried with latent space vectors. With this architecture, we thus developed a novel framework which can not only build nicely clustered latent spaces based on the structural similarities of molecules but also has the capability of doing some intelligent navigation of those latent spaces to generate some other highly similar molecules.

### 4.6. Base Decoder

This final component of our architecture, the base decoder, consists of a transformer decoder network, a final linear layer, and a softmax layer. The transformer decoder network adds one more block of multi-head attention, which takes in the attention vectors K and V from the output of the unprojection. Moreover, the masking mechanism is infused in the first attention block to mask the 1 position shifted right output embedding. With this, the model is only allowed to take into consideration the feature embeddings from the previous positions. Then the final linear layer is a simple neural network to convert position vector outputs from the transformer decoder network into a logit vector which is then followed by softmax layer to convert this array of logit values into a probability score, and the atom or bond corresponding to the index with highest probability is produced as an output. Once the complete sequence of molecules is generated, it is compared with the original input sequence with cross-entropy as a loss function.

### 4.7. Default Settings

We referred to the first dataset of ~5 k molecules containing natural products, drugs, fluorophores, and metabolites as SI1 and that of ~150 k natural product molecules as SI2.

For both the datasets, the choice of optimizer was Adam [183], the learning rate was 10^−5^, and dropout [184] was 20%. Our model has 4 encoder and decoder blocks and each transformer block has 4 attention heads in its multi-head attention layer. For the SI1 dataset, the maximum sequence length of the molecule (in its SMILES encoding) was found to be of length 412. Therefore, we chose the optimal input sequence length post data preprocessing to be 450. The vocabulary size was 79, and the dmodel was set to 64. With these settings the total number of parameters in our model was 342,674, and we chose the maximum possible batch-size to fit on our GPU set-up, which was 40. We randomly split the dataset in the ratio 3:2 for training and validation. However, in this particular scenario we augment the canonical SMILES and train *only* on the augmented SMILES. Our model was shown none of the original canonical SMILES during training and validation. Canonical SMILES were used only for obtaining the projection vectors during testing and the analyses of the latent space.

For the SI2 dataset, the maximum molecule length was 619, and therefore we chose to train the model with input sequence length of 650. The total vocabulary size of the dataset was 69. The dimensionality dmodel of the model was varied for this dataset from around 48 to 256. For most of our analysis, however, we choose 256 dimensional latent space or d_model_ = 256. Therefore, we focused on the settings for this case only. The batch size was set to 20, and the model had a total of 4,678,864 training parameters. In this case, the dataset was split such that 125,000 molecules were used for training and 25,000 reserved for validation.

## 5. Conclusions

The combination of transformers, contrastive learning, and an autoencoder head allows the production of a powerful and disentangled learning system that we have applied to the problem of small molecule similarity. It also admitted a clear understanding of the sparseness with which the space was populated even by over 150,000 molecules, giving optimism that these methods, when scaled to greater numbers of molecules, can learn many molecular properties of interest to the computational chemical biologist.

## References

[1] LeCun Y., Bengio Y., Hinton G. (2015). Deep learning. Nature.

[2] Schmidhuber J. (2015). Deep learning in neural networks: An overview. Neural Netw..

[3] Brown T.B., Mann B., Ryder N., Subbiah M., Kaplan J., Dhariwal P., Neelakantan A., Shyam P., Sastry G., Askell A. (2020). Language models are few-shot learners. arXiv.

[4] Senior A.W., Evans R., Jumper J., Kirkpatrick J., Sifre L., Green T., Qin C., Zidek A., Nelson A.W.R., Bridgland A. (2020). Improved protein structure prediction using potentials from deep learning. Nature.

[5] Samanta S., O’Hagan S., Swainston N., Roberts T.J., Kell D.B. (2020). VAE-Sim: A novel molecular similarity measure based on a variational autoencoder. Molecules.

[6] Kingma D., Welling M. (2014). Auto-encoding variational Bayes. arXiv.

[7] Kingma D.P., Welling M. (2019). An introduction to variational autoencoders. arXiv.

[8] Wei R., Mahmood A. (2021). Recent advances in variational autoencoders with representation learning for biomedical informatics: A survey. IEEE Access.

[9] Wei R., Garcia C., El-Sayed A., Peterson V., Mahmood A. (2020). Variations in variational autoencoders—A comparative evaluation. IEEE Access.

[10] Van Deursen R., Tetko I.V., Godin G. (2020). Beyond chemical 1d knowledge using transformers. arXiv.

[11] Vaswani A., Shazeer N., Parmar N., Uszkoreit J., Jones L., Gomez A.N., Kaiser L., Polosukhin I. (2017). Attention is all you need. arXiv.

[12] Chithrananda S., Grand G., Ramsundar B. (2020). Chemberta: Large-scale self-supervised pretraining for molecular property prediction. arXiv.

[13] Sundararajan M., Taly A., Yan Q. (2017). Axiomatic attribution for deep networks. arXiv.

[14] Simonyan K., Vedaldi A., Zisserman A. (2013). Deep inside convolutional networks: Visualising image classification models and saliency maps. arXiv.

[15] Azodi C.B., Tang J., Shiu S.H. (2020). Opening the black box: Interpretable machine learning for geneticists. Trends Genet..

[16] Core M.G., Lane H.C., van Lent M., Gomboc D., Solomon S., Rosenberg M. (2006). Building explainable artificial intelligence systems. AAAI.

[17] Holzinger A., Biemann C., Pattichis C.S., Kell D.B. (2017). What do we need to build explainable AI systems for the medical domain?. arXiv.

[18] Samek W., Montavon G., Vedaldi A., Hansen L.K., Müller K.-R. (2019). Explainable AI: Interpreting, Explaining and Visualizing Deep Learning.

[19] Singh A., Sengupta S., Lakshminarayanan V. (2020). Explainable deep learning models in medical image analysis. arXiv.

[20] Tjoa E., Guan C. (2019). A survey on explainable artificial intelligence (XAI): Towards medical XAI. arXiv.

[21] Arrieta A.B., Díaz-Rodríguez N., Del Ser J., Bennetot A., Tabik S., Barbado A., Garcia S., Gil-Lopez S., Molina D., Benjamins R. (2020). Explainable artificial intelligence (XAI): Concepts, taxonomies, opportunities and challenges toward responsible AI. Inf. Fusion.

[22] Gunning D., Stefik M., Choi J., Miller T., Stumpf S., Yang G.Z. (2019). XAI-explainable artificial intelligence. Sci. Robot..

[23] Parmar G., Li D., Lee K., Tu Z. (2020). Dual contradistinctive generative autoencoder. arXiv.

[24] Peis I., Olmos P.M., Artés-Rodríguez A. (2020). Unsupervised learning of global factors in deep generative models. arXiv.

[25] Klys J., Snell J., Zemel R. (2018). Learning latent subspaces in variational autoencoders. arXiv.

[26] He Z., Kan M., Zhang J., Shan S. (2020). PA-GAN: Progressive attention generative adversarial network for facial attribute editing. arXiv.

[27] Shen X., Liu F., Dong H., Lian Q., Chen Z., Zhang T. (2020). Disentangled generative causal representation learning. arXiv.

[28] Esser P., Rombach R., Ommer B. (2020). A note on data biases in generative models. arXiv.

[29] Kumar A., Sattigeri P., Balakrishnan A. (2017). Variational inference of disentangled latent concepts from unlabeled observations. arXiv.

[30] Kim H., Mnih A. (2018). Disentangling by factorising. arXiv.

[31] Locatello F., Bauer S., Lucic M., Rätsch G., Gelly S., Schölkopf B., Bachem O. (2018). Challenging common assumptions in the unsupervised learning of disentangled representations. arXiv.

[32] Locatello F., Tschannen M., Bauer S., Rätsch G., Schölkopf B., Bachem O. (2019). Disentangling factors of variation using few labels. arXiv.

[33] Locatello F., Poole B., Rätsch G., Schölkopf B., Bachem O., Tschannen M. (2020). Weakly-supervised disentanglement without compromises. arXiv.

[34] Oldfield J., Panagakis Y., Nicolaou M.A. (2021). Adversarial learning of disentangled and generalizable representations of visual attributes. IEEE Trans. Neural Netw. Learn. Syst..

[35] Pandey A., Schreurs J., Suykens J.A.K. (2021). Generative restricted kernel machines: A framework for multi-view generation and disentangled feature learning. Neural Netw..

[36] Hao Z., Lv D., Li Z., Cai R., Wen W., Xu B. (2021). Semi-supervised disentangled framework for transferable named entity recognition. Neural Netw..

[37] Shen Y., Yang C., Tang X., Zhou B. (2020). Interfacegan: Interpreting the disentangled face representation learned by gans. IEEE Trans. Pattern Anal. Mach. Intell..

[38] Tang Y., Tang Y., Zhu Y., Xiao J., Summers R.M. (2021). A disentangled generative model for disease decomposition in chest x-rays via normal image synthesis. Med. Image Anal..

[39] Cootes T.F., Edwards G.J., Taylor C.J. (2001). Active appearance models. IEEE Trans. Pattern Anal. Mach. Intell..

[40] Cootes T.F., Taylor C.J., Cooper D.H., Graham J. (1995). Active shape models—Their training and application. Comput. Vis. Image Underst..

[41] Hill A., Cootes T.F., Taylor C.J. (1996). Active shape models and the shape approximation problem. Image Vis. Comput..

[42] Salam H., Seguier R. (2018). A survey on face modeling: Building a bridge between face analysis and synthesis. Vis. Comput..

[43] Bozkurt A., Esmaeili B., Brooks D.H., Dy J.G., van de Meent J.-W. (2019). Evaluating combinatorial generalization in variational autoencoders. arXiv.

[44] Alemi A.A., Poole B., Fischer I., Dillon J.V., Saurous R.A., Murphy K. (2019). Fixing a broken ELBO. arXiv.

[45] Zhao S., Song J., Ermon S. (2017). InfoVAE: Balancing learning and inference in variational autoencoders. arXiv.

[46] Leibfried F., Dutordoir V., John S.T., Durrande N. (2020). A tutorial on sparse Gaussian processes and variational inference. arXiv.

[47] Rezende D.J., Viola F. (2018). Taming VAEs. arXiv.

[48] Dai B., Wipf D. (2019). Diagnosing and enhancing VAE models. arXiv.

[49] Li Y., Yu S., Principe J.C., Li X., Wu D. (2020). PRI-VAE: Principle-of-relevant-information variational autoencoders. arXiv.

[50] Higgins I., Matthey L., Pal A., Burgess C., Glorot X., Botvinick M., Mohamed S., Lerchner A. β-VAE: Learning basic visual concepts with a constrained variational framework. Proceedings of the ICLR 2017.

[51] Burgess C.P., Higgins I., Pal A., Matthey L., Watters N., Desjardins G., Lerchner A. (2018). Understanding disentangling in β-VAE. arXiv.

[52] Havtorn J.D., Frellsen J., Hauberg S., Maaløe L. (2021). Hierarchical vaes know what they don’t know. arXiv.

[53] Kumar A., Poole B. (2021). On implicit regularization in β-VAEs. arXiv.

[54] Yang T., Ren X., Wang Y., Zeng W., Zheng N., Ren P. (2021). GroupifyVAE: From group-based definition to VAE-based unsupervised representation disentanglement. arXiv.

[55] Gatopoulos I., Tomczak J.M. (2020). Self-supervised variational auto-encoders. arXiv.

[56] Rong Y., Bian Y., Xu T., Xie W., Wei Y., Huang W., Huang J. (2020). Self-supervised graph transformer on large-scale molecular data. arXiv.

[57] Saeed A., Grangier D., Zeghidour N. (2020). Contrastive learning of general-purpose audio representations. arXiv.

[58] Aneja J., Schwing A., Kautz J., Vahdat A. (2020). NCP-VAE: Variational autoencoders with noise contrastive priors. arXiv.

[59] Artelt A., Hammer B. (2020). Efficient computation of contrastive explanations. arXiv.

[60] Ciga O., Martel A.L., Xu T. (2020). Self supervised contrastive learning for digital histopathology. arXiv.

[61] Chen T., Kornblith S., Norouzi M., Hinton G. (2020). A simple framework for contrastive learning of visual representations. arXiv.

[62] Jaiswal A., Babu A.R., Zadeh M.Z., Banerjee D., Makedon F. (2020). A survey on contrastive self-supervised learning. arXiv.

[63] Purushwalkam S., Gupta A. (2020). Demystifying contrastive self-supervised learning: Invariances, augmentations and dataset biases. arXiv.

[64] Van den Oord A., Li Y., Vinyals O. (2018). Representation learning with contrastive predictive coding. arXiv.

[65] Verma V., Luong M.-T., Kawaguchi K., Pham H., Le Q.V. (2020). Towards domain-agnostic contrastive learning. arXiv.

[66] Le-Khac P.H., Healy G., Smeaton A.F. (2020). Contrastive representation learning: A framework and review. arXiv.

[67] Wang Q., Meng F., Breckon T.P. (2020). Data augmentation with norm-VAE for unsupervised domain adaptation. arXiv.

[68] Li H., Zhang X., Sun R., Xiong H., Tian Q. (2020). Center-wise local image mixture for contrastive representation learning. arXiv.

[69] You Y., Chen T., Sui Y., Chen T., Wang Z., Shen Y. (2020). Graph contrastive learning with augmentations. arXiv.

[70] Willett P. (2011). Similarity-based data mining in files of two-dimensional chemical structures using fingerprint measures of molecular resemblance. Wires Data Min. Knowl..

[71] Stumpfe D., Bajorath J. (2011). Similarity searching. Wires Comput. Mol. Sci..

[72] Maggiora G., Vogt M., Stumpfe D., Bajorath J. (2014). Molecular similarity in medicinal chemistry. J. Med. Chem..

[73] Irwin J.J., Shoichet B.K. (2005). ZINC--a free database of commercially available compounds for virtual screening. J. Chem. Inf. Model..

[74] Ertl P., Schuffenhauer A. (2009). Estimation of synthetic accessibility score of drug-like molecules based on molecular complexity and fragment contributions. J. Cheminform..

[75] Patel H., Ihlenfeldt W.D., Judson P.N., Moroz Y.S., Pevzner Y., Peach M.L., Delannee V., Tarasova N.I., Nicklaus M.C. (2020). Savi, in silico generation of billions of easily synthesizable compounds through expert-system type rules. Sci. Data.

[76] Bickerton G.R., Paolini G.V., Besnard J., Muresan S., Hopkins A.L. (2012). Quantifying the chemical beauty of drugs. Nat. Chem..

[77] Cernak T., Dykstra K.D., Tyagarajan S., Vachal P., Krska S.W. (2016). The medicinal chemist’s toolbox for late stage functionalization of drug-like molecules. Chem. Soc. Rev..

[78] Lovrić M., Molero J.M., Kern R. (2019). PySpark and RDKit: Moving towards big data in cheminformatics. Mol. Inform..

[79] Clyde A., Ramanathan A., Stevens R. (2021). Scaffold embeddings: Learning the structure spanned by chemical fragments, scaffolds and compounds. arXiv.

[80] Arús-Pous J., Awale M., Probst D., Reymond J.L. (2019). Exploring chemical space with machine learning. Chem. Int. J. Chem..

[81] Awale M., Probst D., Reymond J.L. (2017). WebMolCS: A web-based interface for visualizing molecules in three-dimensional chemical spaces. J. Chem. Inf. Model..

[82] Baldi P., Muller K.R., Schneider G. (2011). Charting chemical space: Challenges and opportunities for artificial intelligence and machine learning. Mol. Inform..

[83] Chen Y., Garcia de Lomana M., Friedrich N.O., Kirchmair J. (2018). Characterization of the chemical space of known and readily obtainable natural products. J. Chem. Inf. Model..

[84] Drew K.L.M., Baiman H., Khwaounjoo P., Yu B., Reynisson J. (2012). Size estimation of chemical space: How big is it?. J. Pharm. Pharmacol..

[85] Ertl P. (2014). Visualization of chemical space for medicinal chemists. J. Cheminform..

[86] Gonzalez-Medina M., Prieto-Martinez F.D., Naveja J.J., Mendez-Lucio O., El-Elimat T., Pearce C.J., Oberlies N.H., Figueroa M., Medina-Franco J.L. (2016). Chemoinformatic expedition of the chemical space of fungal products. Future Med. Chem..

[87] Klimenko K., Marcou G., Horvath D., Varnek A. (2016). Chemical space mapping and structure-activity analysis of the chembl antiviral compound set. J. Chem. Inf. Model..

[88] Lin A., Horvath D., Afonina V., Marcou G., Reymond J.L., Varnek A. (2018). Mapping of the available chemical space versus the chemical universe of lead-like compounds. ChemMedChem.

[89] Lucas X., Gruning B.A., Bleher S., Günther S. (2015). The purchasable chemical space: A detailed picture. J. Chem. Inf. Model..

[90] Nigam A., Friederich P., Krenn M., Aspuru-Guzik A. (2019). Augmenting genetic algorithms with deep neural networks for exploring the chemical space. arXiv.

[91] O’Hagan S., Kell D.B. (2019). Generation of a small library of natural products designed to cover chemical space inexpensively. Pharm. Front..

[92] Polishchuk P.G., Madzhidov T.I., Varnek A. (2013). Estimation of the size of drug-like chemical space based on GDB-17 data. J. Comput. Aided Mol. Des..

[93] Reymond J.L. (2015). The chemical space project. Acc. Chem. Res..

[94] Rosén J., Gottfries J., Muresan S., Backlund A., Oprea T.I. (2009). Novel chemical space exploration via natural products. J. Med. Chem..

[95] Thakkar A., Selmi N., Reymond J.L., Engkvist O., Bjerrum E. (2020). ‘Ring breaker’: Neural network driven synthesis prediction of the ring system chemical space. J. Med. Chem..

[96] Thiede L.A., Krenn M., Nigam A., Aspuru-Guzik A. (2020). Curiosity in exploring chemical space: Intrinsic rewards for deep molecular reinforcement learning. arXiv.

[97] Coley C.W. (2021). Defining and exploring chemical spaces. Trends Chem..

[98] Bender A., Glen R.C. (2004). Molecular similarity: A key technique in molecular informatics. Org. Biomol. Chem..

[99] O’Hagan S., Kell D.B. (2017). Consensus rank orderings of molecular fingerprints illustrate the ‘most genuine’ similarities between marketed drugs and small endogenous human metabolites, but highlight exogenous natural products as the most important ‘natural’ drug transporter substrates. ADMET DMPK.

[100] Sterling T., Irwin J.J. (2015). ZINC 15—Ligand discovery for everyone. J. Chem. Inf. Model..

[101] Raffel C., Shazeer N., Roberts A., Lee K., Narang S., Matena M., Zhou Y., Li W., Liu P.J. (2019). Exploring the limits of transfer learning with a unified text-to-text transformer. arXiv.

[102] Rives A., Goyal S., Meier J., Guo D., Ott M., Zitnick C.L., Ma J., Fergus R. (2019). Biological structure and function emerge from scaling unsupervised learning to 250 million protein sequences. bioRxiv.

[103] So D.R., Liang C., Le Q.V. (2019). The evolved transformer. arXiv.

[104] Grechishnikova D. (2020). Transformer neural network for protein specific *de novo* drug generation as machine translation problem. bioRxiv.

[105] Choromanski K., Likhosherstov V., Dohan D., Song X., Gane A., Sarlos T., Hawkins P., Davis J., Mohiuddin A., Kaiser L. (2020). Rethinking attention with Performers. arXiv.

[106] Yun C., Bhojanapalli S., Rawat A.S., Reddi S.J., Kumar S. (2019). Are transformers universal approximators of sequence-to-sequence functions?. arXiv.

[107] Dosovitskiy A., Beyer L., Kolesnikov A., Weissenborn D., Zhai X., Unterthiner T., Dehghani M., Minderer M., Heigold G., Gelly S. (2020). An image is worth 16 × 16 words: Transformers for image recognition at scale. arXiv.

[108] Fedus W., Zoph B., Shazeer N. (2021). Switch transformers: Scaling to trillion parameter models with simple and efficient sparsity. arXiv.

[109] Lu K., Grover A., Abbeel P., Mordatch I. (2021). Pretrained transformers as universal computation engines. arXiv.

[110] Goyal P., Caron M., Lefaudeux B., Xu M., Wang P., Pai V., Singh M., Liptchinsky V., Misra I., Joulin A. (2021). Self-supervised pretraining of visual features in the wild. arXiv.

[111] Wang Y., Wang J., Cao Z., Farimani A.B. (2021). MolCLR: Molecular contrastive learning of representations via graph neural networks. arXiv.

[112] Chen T., Kornblith S., Swersky K., Norouzi M., Hinton G. (2020). Big self-supervised models are strong semi-supervised learners. arXiv.

[113] O’Hagan S., Kell D.B. (2020). Structural similarities between some common fluorophores used in biology, marketed drugs, endogenous metabolites, and natural products. Mar. Drugs.

[114] Ji Z., Zou X., Huang T., Wu S. (2020). Unsupervised few-shot feature learning via self-supervised training. Front. Comput. Neurosci..

[115] Wang Y., Yao Q., Kwok J., Ni L.M. (2019). Generalizing from a few examples: A survey on few-shot learning. arXiv.

[116] Ma J., Fong S.H., Luo Y., Bakkenist C.J., Shen J.P., Mourragui S., Wessels L.F.A., Hafner M., Sharan R., Peng J. (2021). Few-shot learning creates predictive models of drug response that translate from high-throughput screens to individual patients. Nat. Cancer.

[117] Li F.-F., Fergus R., Perona P. (2006). One-shot learning of object categories. IEEE Trans. Pattern Anal. Mach. Intell..

[118] Rezende D.J., Mohamed S., Danihelka I., Gregor K., Wierstra D. (2016). One-shot generalization in deep generative models. arXiv.

[119] Altae-Tran H., Ramsundar B., Pappu A.S., Pande V. (2017). Low data drug discovery with one-shot learning. ACS Cent. Sci..

[120] Baskin I.I. (2019). Is one-shot learning a viable option in drug discovery?. Expert Opin. Drug Discov..

[121] He X., Zhao K.Y., Chu X.W. (2021). AutoML: A survey of the state-of-the-art. Knowl. Based Syst..

[122] Chochlakis G., Georgiou E., Potamianos A. (2021). End-to-end generative zero-shot learning via few-shot learning. arXiv.

[123] Majumder O., Ravichandran A., Maji S., Polito M., Bhotika R., Soatto S. (2021). Revisiting contrastive learning for few-shot classification. arXiv.

[124] Dasari S., Gupta A. (2020). Transformers for one-shot visual imitation. arXiv.

[125] Logeswaran L., Lee A., Ott M., Lee H., Ranzato M.A., Szlam A. (2020). Few-shot sequence learning with transformers. arXiv.

[126] Belkin M., Hsu D., Ma S., Mandal S. (2019). Reconciling modern machine-learning practice and the classical bias-variance trade-off. Proc. Natl. Acad. Sci. USA.

[127] Van der Maaten L., Hinton G. (2008). Visualizing data using t-sne. J. Mach. Learn. Res..

[128] Van der Maaten L. (2009). Learning a parametric embedding by preserving local structure. Proc. AISTATS.

[129] McInnes L., Healy J., Melville J. (2018). UMAP: Uniform manifold approximation and projection for dimension reduction. arXiv.

[130] McInnes L., Healy J., Saul N., Großberger L. (2018). UMAP: Uniform manifold approximation and projection. J. Open Source Softw..

[131] Dickens D., Rädisch S., Chiduza G.N., Giannoudis A., Cross M.J., Malik H., Schaeffeler E., Sison-Young R.L., Wilkinson E.L., Goldring C.E. (2018). Cellular uptake of the atypical antipsychotic clozapine is a carrier-mediated process. Mol. Pharm..

[132] Horvath D., Jeandenans C. (2003). Neighborhood behavior of in silico structural spaces with respect to in vitro activity spaces-a novel understanding of the molecular similarity principle in the context of multiple receptor binding profiles. J. Chem. Inf. Comput. Sci..

[133] Bender A., Jenkins J.L., Li Q.L., Adams S.E., Cannon E.O., Glen R.C. (2006). Molecular similarity: Advances in methods, applications and validations in virtual screening and qsar. Annu. Rep. Comput. Chem..

[134] Horvath D., Koch C., Schneider G., Marcou G., Varnek A. (2011). Local neighborhood behavior in a combinatorial library context. J. Comput. Aid. Mol. Des..

[135] Gasteiger J. (2003). Handbook of Chemoinformatics: From Data to Knowledge.

[136] Bajorath J. (2004). Chemoinformatics: Concepts, Methods and Tools for Drug Discovery.

[137] Sutherland J.J., Raymond J.W., Stevens J.L., Baker T.K., Watson D.E. (2012). Relating molecular properties and in vitro assay results to in vivo drug disposition and toxicity outcomes. J. Med. Chem..

[138] Capecchi A., Probst D., Reymond J.L. (2020). One molecular fingerprint to rule them all: Drugs, biomolecules, and the metabolome. J. Cheminform..

[139] Muegge I., Mukherjee P. (2016). An overview of molecular fingerprint similarity search in virtual screening. Expert Opin. Drug Discov..

[140] Nisius B., Bajorath J. (2010). Rendering conventional molecular fingerprints for virtual screening independent of molecular complexity and size effects. ChemMedChem.

[141] Riniker S., Landrum G.A. (2013). Similarity maps—A visualization strategy for molecular fingerprints and machine-learning methods. J. Cheminform..

[142] Vogt I., Stumpfe D., Ahmed H.E., Bajorath J. (2007). Methods for computer-aided chemical biology. Part 2: Evaluation of compound selectivity using 2d molecular fingerprints. Chem. Biol. Drug Des..

[143] O’Hagan S., Swainston N., Handl J., Kell D.B. (2015). A ‘rule of 0.5′ for the metabolite-likeness of approved pharmaceutical drugs. Metabolomics.

[144] O’Hagan S., Kell D.B. (2015). Understanding the foundations of the structural similarities between marketed drugs and endogenous human metabolites. Front. Pharm..

[145] O’Hagan S., Kell D.B. (2015). The apparent permeabilities of Caco-2 cells to marketed drugs: Magnitude, and independence from both biophysical properties and endogenite similarities. Peer J..

[146] O’Hagan S., Kell D.B. (2016). MetMaxStruct: A Tversky-similarity-based strategy for analysing the (sub)structural similarities of drugs and endogenous metabolites. Front. Pharm..

[147] O’Hagan S., Kell D.B. (2017). Analysis of drug-endogenous human metabolite similarities in terms of their maximum common substructures. J. Cheminform..

[148] O’Hagan S., Kell D.B. (2018). Analysing and navigating natural products space for generating small, diverse, but representative chemical libraries. Biotechnol. J..

[149] Gawehn E., Hiss J.A., Schneider G. (2016). Deep learning in drug discovery. Mol. Inform..

[150] Gómez-Bombarelli R., Wei J.N., Duvenaud D., Hernández-Lobato J.M., Sánchez-Lengeling B., Sheberla D., Aguilera-Iparraguirre J., Hirzel T.D., Adams R.P., Aspuru-Guzik A. (2018). Automatic chemical design using a data-driven continuous representation of molecules. ACS Cent. Sci..

[151] Sanchez-Lengeling B., Aspuru-Guzik A. (2018). Inverse molecular design using machine learning: Generative models for matter engineering. Science.

[152] Arús-Pous J., Probst D., Reymond J.L. (2018). Deep learning invades drug design and synthesis. Chimia.

[153] Yang K., Swanson K., Jin W., Coley C., Eiden P., Gao H., Guzman-Perez A., Hopper T., Kelley B., Mathea M. (2019). Analyzing learned molecular representations for property prediction. J. Chem. Inf. Model..

[154] Zhavoronkov A., Ivanenkov Y.A., Aliper A., Veselov M.S., Aladinskiy V.A., Aladinskaya A.V., Terentiev V.A., Polykovskiy D.A., Kuznetsov M.D., Asadulaev A. (2019). Deep learning enables rapid identification of potent DDR1 kinase inhibitors. Nat. Biotechnol..

[155] Khemchandani Y., O’Hagan S., Samanta S., Swainston N., Roberts T.J., Bollegala D., Kell D.B. (2020). DeepGraphMolGen, a multiobjective, computational strategy for generating molecules with desirable properties: A graph convolution and reinforcement learning approach. J. Cheminform..

[156] Shen C., Krenn M., Eppel S., Aspuru-Guzik A. (2020). Deep molecular dreaming: Inverse machine learning for de-novo molecular design and interpretability with surjective representations. arXiv.

[157] Moret M., Friedrich L., Grisoni F., Merk D., Schneider G. (2020). Generative molecular design in low data regimes. Nat. Mach. Intell..

[158] Kell D.B., Samanta S., Swainston N. (2020). Deep learning and generative methods in cheminformatics and chemical biology: Navigating small molecule space intelligently. Biochem. J..

[159] Walters W.P., Barzilay R. (2021). Applications of deep learning in molecule generation and molecular property prediction. Acc. Chem. Res..

[160] Zaheer M., Guruganesh G., Dubey A., Ainslie J., Alberti C., Ontanon S., Pham P., Ravula A., Wang Q., Yang L. (2020). Big bird: Transformers for longer sequences. arXiv.

[161] Hutson M. (2021). The language machines. Nature.

[162] Topal M.O., Bas A., van Heerden I. (2021). Exploring transformers in natural language generation: GPT, BERT, and XLNET. arXiv.

[163] Zandie R., Mahoor M.H. (2021). Topical language generation using transformers. arXiv.

[164] Weininger D. (1988). Smiles, a chemical language and information system.1. Introduction to methodology and encoding rules. J. Chem. Inf. Comput. Sci..

[165] Tetko I.V., Karpov P., Van Deursen R., Godin G. (2020). State-of-the-art augmented NLP transformer models for direct and single-step retrosynthesis. Nat. Commun..

[166] Lim S., Lee Y.O. (2020). Predicting chemical properties using self-attention multi-task learning based on SMILES representation. arXiv.

[167] Pflüger P.M., Glorius F. (2020). Molecular machine learning: The future of synthetic chemistry?. Angew. Chem. Int. Ed. Engl..

[168] Shin B., Park S., Bak J., Ho J.C. (2020). Controlled molecule generator for optimizing multiple chemical properties. arXiv.

[169] Liu X., Zhang F., Hou Z., Mian L., Wang Z., Zhang J., Tang J. (2020). Self-supervised learning: Generative or contrastive. arXiv.

[170] Wanyan T., Honarvar H., Jaladanki S.K., Zang C., Naik N., Somani S., Freitas J.K.D., Paranjpe I., Vaid A., Miotto R. (2021). Contrastive learning improves critical event prediction in COVID-19 patients. arXiv.

[171] Kostas D., Aroca-Ouellette S., Rudzicz F. (2021). Bendr: Using transformers and a contrastive self-supervised learning task to learn from massive amounts of EEG data. arXiv.

[172] Everitt B.S. (1993). Cluster Analysis.

[173] Botvinick M., Barrett D.G.T., Battaglia P., de Freitas N., Kumaran D., Leibo J.Z., Lillicrap T., Modayil J., Mohamed S., Rabinowitz N.C. (2017). Building machines that learn and think for themselves. Behav. Brain Sci..

[174] Hassabis D., Kumaran D., Summerfield C., Botvinick M. (2017). Neuroscience-inspired artificial intelligence. Neuron.

[175] Shevlin H., Vold K., Crosby M., Halina M. (2019). The limits of machine intelligence despite progress in machine intelligence, artificial general intelligence is still a major challenge. EMBO Rep..

[176] Pei J., Deng L., Song S., Zhao M., Zhang Y., Wu S., Wang G., Zou Z., Wu Z., He W. (2019). Towards artificial general intelligence with hybrid Tianjic chip architecture. Nature.

[177] Stanley K.O., Clune J., Lehman J., Miikkulainen R. (2019). Designing neural networks through neuroevolution. Nat. Mach. Intell..

[178] Zhang Y., Qu P., Ji Y., Zhang W., Gao G., Wang G., Song S., Li G., Chen W., Zheng W. (2020). A system hierarchy for brain-inspired computing. Nature.

[179] Nadji-Tehrani M., Eslami A. (2020). A brain-inspired framework for evolutionary artificial general intelligence. IEEE Trans. Neural Netw. Learn. Syst..

[180] Bjerrum E.J. (2017). SMILES enumeration as data augmentation for neural network modeling of molecules. arXiv.

[181] Sohn K. (2016). Improved deep metric learning with multi-class n-pair loss objective. NIPS.

[182] Wu Z., Xiong Y., Yu S., Lin D. (2018). Unsupervised feature learning via non-parametric instance-level discrimination. arXiv.

[183] Kingma D.P., Ba J.L. (2015). Adam: A method for stochastic optimization. arXiv.

[184] Srivastava N., Hinton G., Krizhevsky A., Sutskever I., Salakhutdinov R. (2014). Dropout: A simple way to prevent neural networks from overfitting. J. Mach. Learn. Res..

